# A ‘Simple Anterior Fish Excluder’ (SAFE) for Mitigating Penaeid-Trawl Bycatch

**DOI:** 10.1371/journal.pone.0123124

**Published:** 2015-04-02

**Authors:** Matthew J. McHugh, Matt K. Broadhurst, David J. Sterling, Russell B. Millar

**Affiliations:** 1 Marine and Estuarine Ecology Unit, School of Biological Sciences, University of Queensland, Brisbane, Queensland, Australia; 2 NSW Department of Primary Industries, Fisheries Conservation Technology Unit, Coffs Harbour, New South Wales, Australia; 3 Sterling Trawl Gear Services, Manly, Queensland, Australia; 4 Department of Statistics, The University of Auckland, Auckland, New Zealand; Hellenic Centre for Marine Research, GREECE

## Abstract

Various plastic strips and sheets (termed ‘simple anterior fish excluders’−SAFEs) were positioned across the openings of penaeid trawls in attempts at reducing the unwanted bycatches of small teleosts. Initially, three SAFEs (a single wire without, and with small and large plastic panels) were compared against a control (no SAFE) on paired beam trawls. All SAFEs maintained targeted *Metapenaeus macleayi* catches, while the largest plastic SAFE significantly reduced total bycatch by 51% and the numbers of *Pomatomus saltatrix*, *Mugil cephalus* and *Herklotsichthys castelnaui* by up to 58%. A redesigned SAFE (‘continuous plastic’) was subsequently tested (against a control) on paired otter trawls, significantly reducing total bycatch by 28% and *P*. *saltatrix* and *H*. *castelnaui* by up to 42%. The continuous-plastic SAFE also significantly reduced *M*. *macleayi* catches by ~7%, but this was explained by ~5% less wing-end spread, and could be simply negated through otter-board refinement. Further work is required to refine the tested SAFEs, and to quantify species-specific escape mechanisms. Nevertheless, the SAFE concept might represent an effective approach for improving penaeid-trawl selectivity.

## Introduction

The capture and mortality of unwanted organisms (termed ‘bycatch’) by mobile demersal fishing gears is a global issue affecting many fisheries [1]. This is especially the case for penaeid trawling, which despite contributing only ~1.5% towards the total global marine wild harvest (estimated at a plateau of ~80 m t since 1985 [2]), accounts for >25% of all discarded bycatch (~7.3 m t per annum [1]); typically comprising small teleosts (<20 cm total length−TL), crustaceans and cephalopods [1, 3]. Historically, the mortality of such discards has raised wide-spread concerns, and primarily because of the potential for deleterious impacts on subsequent stocks [4, 5].

Considerable research has been done to mitigate penaeid-trawl bycatch and associated mortalities [6, 7]. Beyond temporal and spatial closures [7] the greatest efforts have focussed on retrospectively fitting ‘bycatch reduction devices’ (BRDs) to existing trawls. Broadly, such BRDs can be separated into two categories according to their principle separating function: those that rely on species-specific differences in size (termed ‘mechanical-type BRDs’; e.g. the ‘Nordmøre-grid’); or behaviour (‘behavioural-type BRDs’; e.g. strategic ‘square-mesh panels’) to either actively or passively separate catches [6].

Notwithstanding their different classifications, the majority of BRDs are located in the posterior trawl (i.e. codend) and compared to conventional configurations can maintain penaeid catches within a ~10% loss, while reducing unwanted bycatches by ~30−70% [6]. Such results are positive, although there remains very little information on the mortality of organisms escaping BRDs (and therefore their ultimate benefit); primarily because accurate assessments are difficult, if not impossible, for many fisheries [8, 9]. However, because BRDs that facilitate the rapid escape of organisms with minimal physical contact (e.g. behavioural-type designs) should evoke low mortalities, an even more appropriate concept might be to anteriorly locate designs, and so promote complete trawl avoidance.

While the widespread use of such anterior BRDs is relatively uncommon, there have been successful attempts at demonstrating their utility [10]. For example, Seidel and Watson [10] designed a ‘fish barrier’, comprising mesh webbing across the trawl mouth that precluded the entry of large organisms, and used electrical stimulation to force penaeids up through an open benthic panel, and into the trawl. However, while this configuration had great potential, subsequently cheaper and more easily adaptable (to existing trawl codends) BRDs might have contributed towards its lack of commercial uptake. Also, some mesh-barrier designs (e.g. seal mitigation devices; [11]), placed at the trawl mouth can clog (e.g. with seaweed), which could either prevent penaeids entering or, alternatively, reduce wing-end spread and the area trawled [12].

The latter issue raises an important consideration. It is well established that complex BRDs are much less likely to be adopted and/or used correctly than those that are inexpensive and/or simple to maintain and operate [6]. Consequently, in terms of reducing unaccounted fishing mortality, the wide-scale use of simple and even marginally effective BRDs ultimately will have greater benefits than the limited use of far more effective designs. Given the above, an alternative to completely physically obstructing the trawl mouth may be to insert a behavioural-type BRD, which although being the less effective category of BRDs [6] should be smaller and less likely to affect trawl performance. While the concept of anterior behavioural-type BRDs is not new (e.g. [12–14]), the difficulty remains in focusing on the stimuli (e.g. visual or auditory) that will elicit the greatest response among non-target individuals without impacting on target species [15].

Irrespective of the BRD location (anterior or posterior) or type (behavioural or mechanical), during development there always should be an emphasis on hypotheses testing within a strong empirical experimental design [16]. To maximise penaeid catches while minimizing bycatch, any modifications should be clearly identified through systematic testing within the full range of possibilities [17, 18]. Methodically assessing modifications will facilitate further testing, acceptance or reassessment if the desired result is not achieved [17].

During a recent study in an Australian penaeid-trawl fishery, we tested an anteriorly located BRD that met some of the technical criteria discussed above [14]. Termed the ‘simple anterior fish excluder’ (SAFE), the design comprised a wire between beam-trawl sleds, from which 200- × 60- × 1-mm plastic strips were hung on universal swivels (allowing spinning). Compared to the control, the trawl with the SAFE reduced the catches of one species, southern herring, *Herklotsichthys castelnaui* by 48%, with minimal effect on catches of the targeted school prawns, *Metapenaeus macleayi*.

Here, we expand on the SAFE concept by first assessing the limits of practicality and effectiveness (including the original SAFE tested by McHugh et al. [14]) within a beam-trawl configuration before using this information to develop a prototype for testing on a more dynamic (i.e. non-rigid spreading mechanism) otter trawl. Specifically, our aims were to (i) test the hypothesis of no differences in the effectiveness of the SAFE area (i.e. 1, 3 and 11% of the two-dimensional opening) on the beam trawl and then, using this information, (ii) design and test an appropriate SAFE for use in otter trawling. The work was done in Australia, but the results have broader implications among other national and international crustacean-trawl fisheries.

## Material and Methods

### Ethics statement

The research was done in Lake Wooloweyah (29°26′ S 153°22′ E) New South Wales (NSW) Australia and in accord with the Department of Primary Industries scientific collection permit (No. P01/0059(A)-2.0). No specific permissions were required for access to Lake Wooloweyah. This study did not involve endangered or protected species, and all fish were returned to the water as soon as practicable, following each trawl deployment. Animal ethics approval for the research was granted by the NSW DPI Animal Care and Ethics Committee (Ref. 08/06). This study complied with all relevant regulations pertaining to the conservation of the surrounding environment and nearby wildlife, as detailed in the scientific collection permit.

### Location and vessel

Two experiments were completed in the Lake (sand and mud substrata ~1–2 m depth) during the Austral summer, 2013 on-board a 10-m double-rigged penaeid trawler (104 kw). The trawler had a global positioning system (Lowrance, HDS5) and two independent sum logs (model: Bronze + Log) to record speed over the ground (SOG) and through the water (STW; both in m s^–1^). Load cells (Amalgamated Instrument Company; model no. PA6139) were configured so that they could be attached to bridles (always deployed to 12 m from paired winches) to measure the combined tension (kgf). The wing-end spreads of relevant otter trawls were obtained using Notus paired wireless sensors (see below). Data from the Notus sensors were received through an omnidirectional hydrophone and logged onto a laptop. All electronic data were recorded every 60 s.

### Experiment 1: testing three different SAFEs on a beam trawl

**Fig 1:**
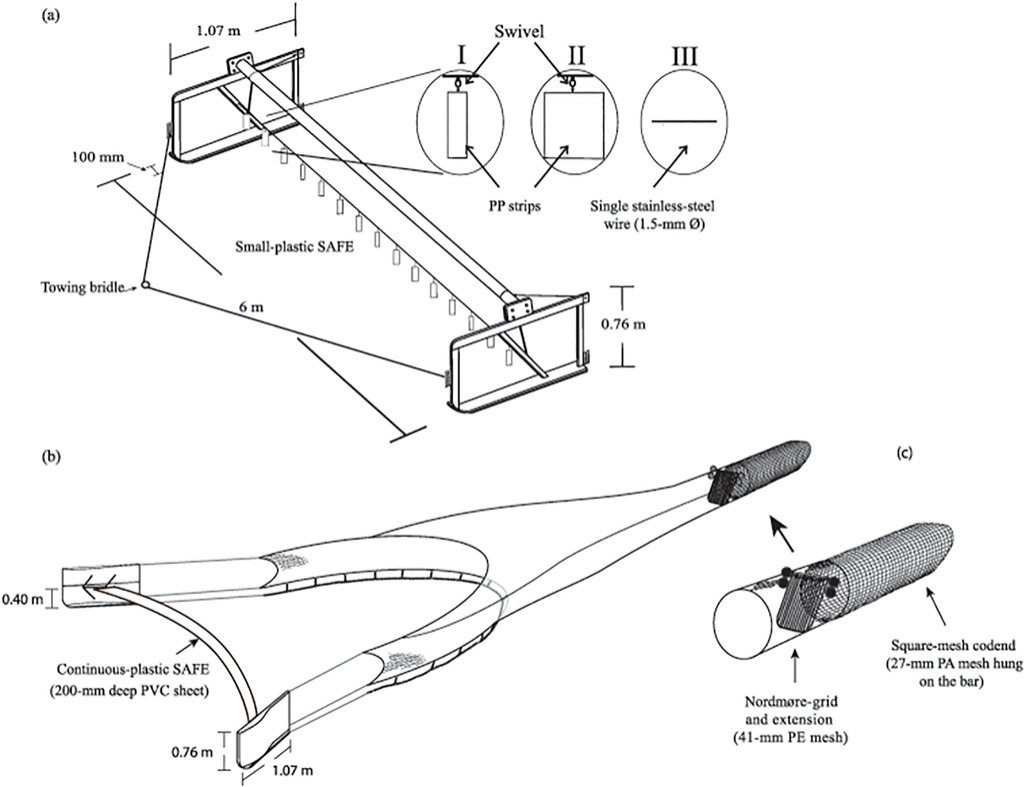
Schematic representation of the (a) beam trawl showing towing bridle and attachment locations of the (I) small-plastic (polypropylene–PP) (60 × 200 × 1 mm), (II) large-plastic (PP) (200 × 200 × 1 mm) and (III) the single-wire (1.50-mm Ø stainless steel) simple anterior fish excluders (SAFEs) tested in experiment 1 and (b) otter trawl with the polyvinyl chloride (PVC) continuous-plastic SAFE tested in experiment 2. The extension (with Nordmøre-grid) and codend (c), used in both experiments, are highlighted. PA, polyamide; PE, polyethylene.

For the first experiment, the trawler was rigged with identical, paired 6-m beam-and-sled (1.07 × 0.76 × 0.1 m; 108 kg) assemblies. These spreading mechanisms were anteriorly attached to the towing wires via a 7.3 m bridle (Fig 1a) and posteriorly to trawl bodies with 9.19 m headlines (and footropes) and constructed from nominal 41-mm mesh (stretched mesh opening–SMO) and 1.25-mm diameter−Ø twisted polyethylene (PE) twine (for a trawl plan, see [14]). Both trawl bodies had identical conventional Nordmøre-grids (28-mm bar spacing) and square-mesh codends (120 × 75 bars) made from nominal 27 mm SMO polyamide mesh (1.25-mm Ø twine) hung on the bar (Fig 1b).

Three SAFE treatments were constructed; all stretched between the sleds at 0.3 above the baseplates (Fig 1a). The first treatment comprised a single 6-m long, 1.50-mm Ø stainless-steel wire (termed ‘single wire’) while the second and third had the same wires, but also included 12 evenly distributed flat strips (all 0.2 m long) of 1-mm thick green polypropylene (PP) that were either 0.06- (termed ‘small plastic’ and the same as those tested by McHugh et al. [14]) or 0.2-m (‘large plastic’) wide (Fig 1a). The PP strips were secured to the main line by a snap-lock (ball bearing) swivel that was attached midway along their leading edges (Fig 1a). Prior to the experiment, the small- and large-plastic strips were secured at several (e.g. centre, edge and middle) attachment points to a pole, which was pulled through the water (at ~1.50 m s^–1^) alongside a wharf and filmed with a Hero 3^+^ GoPro. The plastic strips attached at the centre of their leading edges were observed to spin erratically.

On each fishing day, the paired beams were configured as either the control (i.e. no wire), or with one of the three SAFE treatments and deployed for 40 min. The control and SAFE treatments were then alternated, so that we completed one paired comparison of all four configurations on each day (i.e. six daily deployments). The two trawls were also swapped from side-to-side after the first three deployments, while the load cells were daily rotated from side-to-side. Over seven days, we completed 21 replicate deployments of each SAFE and the control.

### Experiment 2: testing a SAFE on an otter trawl

During the second experiment, the beam trawls were replaced with otter trawls, and the towing wires attached directly to paired cambered otter boards (1.07 × 0.76 m each and a total weight of 108 kg; Fig 1b). Sweep wires (2.89-m) were secured posterior to the otter boards and to 7.35-m headline length trawls that were constructed from the same materials and designs as those in experiment 1 and configured with the exact same Nordmøre-grids and codends (Fig 1b; for a trawl plan see McHugh et al. [14]).

A single SAFE treatment was constructed for use with the otter trawls. Termed the ‘continuous-plastic’, this design comprised a hemmed sheet of flexible white polyvinyl chloride (PVC) measuring 0.2 m wide (same as the green PP strips) × 6.4 m long, through which a 7.25-m (1.50-mm Ø) stainless-steel wire was threaded and terminated in snap clips (Fig 1b). The length of the wire was calculated based on an average wing-end spread during previous testing of the two trawls, and this was extrapolated to derive the otter-board spread [14]. The continuous-plastic SAFE was attached between the otter-board towing points at 0.40 m above the baseplates, so that it extended across the front of the trawl (Fig 1b).

At the start of each fishing day, the Notus paired sensors were attached to the wing ends of the trawls on each side of the vessel. The continuous-plastic SAFE was alternately and randomly clipped in front of one trawl, with both then deployed for 40-min up to six times each day. After three deployments, the trawls were swapped from side-to-side, while the load cells and paired Notus sensors were similarity rotated each day. Over five days, we completed 26 replicate deployments of the control and continuous-plastic SAFE.

### Data collected and statistical analyses

All trawl bodies and codends were checked for mesh uniformity by measuring 15 replicate SMOs using a local, purpose-built gauge. Other technical data collected during each deployment in each experiment included the: (i) warp tension (kgf) for each configuration; (ii) the total distance (m) trawled (sleds on and off the bottom—obtained from the GPS); and (iii) SOG and STW (m s^–1^) (S1 and S2 Tables). Additionally, in experiment 2, data for wing-spread (m) were collected for each deployment (S2 Table).

Biological data collected at the end of each deployment included the: total weights of the targeted *M*. *macleayi* and bycatch; numbers of each bycatch species; and total lengths (TL to the nearest 0.5 cm) of the most abundant teleosts. Random samples of ~500 g of *M*. *macleayi* were bagged and transferred to the laboratory, where they were measured (carapace length—CL in mm), weighed and counted. These latter data were used to estimate the total numbers and the mean CLs caught during each deployment.

The hypothesis of no differences in the mesh sizes within the four trawl bodies, and two extensions and codends was tested in a linear model (LM). Within each experiment, the remaining data were analysed in linear mixed models (LMMs), with some standardised prior to analyses. The numbers and weights of catches were analysed per 40-min deployment and also per ha trawled (calculated using the known beam- and observed otter-trawl wing-end distances and the distance trawled) and as log-transformed data so that predicted effects would be multiplicative. All other data, including the mean CL of *M*. *macleayi*, mean TL per deployment of sufficiently abundant teleosts (occurring in >95% of deployments), drag and area and distance trawled were analysed in their raw form.

All LMMs included ‘anterior-trawl configuration’ (i.e. SAFEs vs. controls) as a fixed effect, while ‘trawls’, ‘sides’, ‘days’ and deployments (within days) were included as random terms. For the LMM assessing drags, ‘load cell’ was included as an additional random term while additional fixed co-variates included ‘SOG’, ‘STW’ (with ‘sum-log’ as a random term) and ‘flow’ (calculated as the speed of the current in the direction of travel and defined as SOG–STW). The preferred models were chosen based on forward variable selection with a p-value of 0.05 required for an effect to enter the model. All models were fitted using either the lmer function from the lme4 package or ASReml in R 2.15.3 (The R Project for Statistical Computing; http://www.r-project.org/), with the significance of anterior-trawl configuration determined using a Wald *F-*value. In experiment 1, any significant Wald *F-*values for anterior-trawl configuration were subsequently explored using the Benjamini-Hochberg-Yekutieli procedure to control the false discovery rate (FDR) for multiple pair-wise comparisons [19].

## Results

There were no significant differences in the SMO between trawl bodies (means ± SE of 41.25 ± 0.08 mm), extensions (41.40 ± 0.17 mm) or codends (27.35 ± 0.10 mm) (LM, *p*>0.05). Pooled across experiments, the trawls caught 1753 and 154 kg of *M*. *macleayi* and total bycatch (Table 1). The total bycatch included 29 species, but in experiment 1, tailor, *Pomatomus saltatrix* (5.5–18.5 cm T), bully mullet, *Mugil cephalus* (5.5–15.5 cm TL), silver biddy, *Gerres subfasciatus* (5.0–13 cm TL), Ramsey’s perchlet, *Ambassis marianus* (3.5–10.5 cm TL), yellowfin bream, *Acanthopagrus australis* (4.0–23.5 cm TL), and southern herring, *H*. *castelnaui* (6.5–16.5 cm TL) comprised >85% of catches (Table 1). In experiment 2, *A*. *marianus* (5.0–13.5 cm TL), *P*. *saltatrix* (4.0–17 cm TL), *G*. *subfasciatus* (6.5–13.5 cm TL), *H*. *castelnaui* (5.5–15 cm TL), *A*. *australis* (5.0–25 cm TL), and tarwhine, *Rhabdosargus sarba* (5.5–11 cm TL) were most prevalent (>77%; Table 1). These seven species, along with *M*. *macleayi*, form the basis of the biological analyses.

**Table 1:** Scientific and common names and numbers (except blue blubber jellyfish, *Catostylus mosaicus*—weights in kg only) of organisms caught during experiments (Exp.) 1 and 2.

*Family*	*Scientific name*	*Common name*	Exp. 1	Exp. 2
*Cnidarians*				
Catostylidae	*Catostylus mosaicus*	Blue blubber jellyfish	108	40
*Crustaceans*				
Palaemonidae	*Macrobrachium novaehollandiae*	Freshwater prawn	2	–
Penaeidae	*Metapenaeus macleayi*	School prawn^1^	584,044	147,116
	*Metapenaeus bennettae*	Green tail prawn^1^	21	49
	*Penaeus monodon*	Tiger prawn^1^	7	3
Portunidae	*Portunus pelagicus*	Blue swimmer crab^1^	6	6
*Elasmobranchs*				
Dasyatididae	*Dasyatis* sp	Stingray	–	1
*Molluscs*				
Loliginidae	*Uroteuthis* sp	Squid^1^	368	201
*Teleosts*				
Ambassidae	*Ambassis jacksoniensis*	Port Jackson glassfish	324	57
	*Ambassis marianus*	Ramsey’s perchlet	470	1,058
Ariidae	*Arius graeffei*	Forktail catfish^1^	1	22
Apogonidae	*Siphamia roseigaster*	Pink-breasted siphonfish	129	65
Carangidae	*Gnathanodon speciosus*	Golden trevally^1^	1	−
	*Pseudocaranx dentex*	Silver trevally^1^	−	1
	*Trachurus novaezelandiae*	Yellowtail scad^1^	1	2
Clupeidae	*Herklotsichthys castelnaui*	Southern herring	400	369
	*Hyperlophus vittatus*	Whitebait	−	5
Engraulidae	*Engraulis australis*	Australian anchovy	45	13
Gerreidae	*Gerres subfasciatus*	Silver biddy	507	447
Hemiramphidae	*Arrhamphus sclerolepis*	Snubnose garfish^1^	3	4
	*Hyporhamphus regularis*	River garfish^1^	16	–
Monodactylidae	*Monodactylus argenteus*	Diamond fish	2	6
Mugilidae	*Liza argentea*	Flat-tail mullet^1^	24	3
	*Mugil cephalus*	Bully mullet^1^	1,046	64
Muraenesocidae	*Muraenesox bagio*	Common pike eel	1	4
Paralichthyidae	*Pseudorhombus arsius*	Largetooth flounder	15	23
Platycephalidae	*Platycephalus fuscus*	Dusky flathead^1^	1	9
Plotosidae	*Euristhmus lepturus*	Longtail catfish	61	175
Pomatomidae	*Pomatomus saltatrix*	Tailor^1^	4,087	937
Scatophagidae	*Selenotoca multifasciata*	Old maid	–	1
Sciaenidae	*Argyrosomus japonicus*	Mulloway^1^	2	2
Sillaginidae	*Sillago ciliata*	Sand whiting^1^	–	2
Soleidae	*Synclidopus macleayanus*	Narrow banded sole	1	−
Sparidae	*Acanthopagrus australis*	Yellowfin bream^1^	420	328
	*Rhabdosargus sarba*	Tarwhine	65	202
Terapontidae	*Pelates quadrilineatus*	Trumpeter^1^	13	4
Tetraodontidae	*Tetractenos glaber*	Toadfish	8	271

−, not present in catches.

^1^economically important

### Experiment 1: testing three different SAFEs on a beam trawl

The four beam-trawl configurations were towed at similar SOGs and STWs (ranging from 1.23 to 1.28 m s^−1^) covering predicted mean ± SE areas between 1.90 ± 0.02 and 1.95 ± 0.02 ha per 40-min deployment, which were not significantly different (LMM, *p*>0.05; Table 2). None of the SAFEs significantly affected drag (predicted means ± SE between 205.3 ± 2.2 and 208.2 ± 2.2 kg, LMM, *p*>0.05; Table 2). STW and SOG were both positively correlated with drag, but they were not statistically significant (*p*>0.05).

**Table 2:** Summaries of Wald *F*-values from linear mixed models assessing the importance of the fixed effect of anterior-trawl configuration (SAFEs vs. controls) in explaining variability among engineering and biological variables.

	Experiment 1	Experiment 2
	Wald *F*	Wald *F*
**Engineering variables**		
Wing-end spread	−	4.19*
Drag	0.62	0.004
Hectare trawled	2.17	22.46***
**Biological variables**		
Wt of *Metapenaeus macleayi* 40 min^–1^	2.53	4.52*
Wt of *M*. *macleayi* ha^–1^	2.56	0.19
No. of *M*. *macleayi* 40 min^−1^	1.56	1.46
No. of *M*. *macleayi* ha^–1^	1.58	0.19
Mean CL of *M*. *macleayi* 40 min^−1^	1.55	5.41*
Wt of fish bycatch 40 min^−1^	22.81***	12.16**
Wt of fish bycatch ha^–1^	23.54***	7.08*
No. of fish bycatch 40 min^−1^	19.18**	9.33**
No. of fish bycatch ha^−1^	19.75***	5.53*
No. of *Pomatomus saltatrix* 40 min^–1^	15.09***	11.93**
No. of *P*. *saltatrix* ha^–1^	17.34***	10.86**
Mean TL of *P*. *saltatrix* 40 min^−1^	1.34	−
No. of *Mugil cephalus* 40 min^–1^	5.06**	−
No. of *M*. *cephalus* ha^–1^	4.99**	−
No. of *Herklotsichthys castelnaui* 40 min^–1^	3.94*	7.00*
No. of *H*. *castelnaui* ha^–1^	3.98*	5.73*
No. of *Gerres subfasciatus* 40 min^–1^	1.49	1.66
No. of *G*. *subfasciatus* ha^–1^	1.24	2.39
No. of *Ambassis marianus* 40 min^–1^	1.77	0.15
No. of *A*. *marianus* ha^–1^	1.45	0.01
No. of *Acanthopagrus australis* 40 min^–1^	1.00	0.11
No. of *A*. *australis* ha^–1^	1.07	0.38
No. of *Rhabdosargus sarba* 40 min^–1^	−	0.25
No. of *R*. *sarba* ha^–1^	−	0.14

Numbers and weights were analysed per 40-min deployment and standardised to per ha trawled calculated using the footrope contact (wing-end spread × distance trawled) and then log-transformed.

−, not relevant.

*p<0.05

**p<0.01

***p<0.001

Because there were no significant differences in the areas trawled, the biological data provided the same interpretations irrespective of standardization (i.e. to per ha; Table 2). Consequently, for convenience (and beyond Table 2), only the catches per 40-min deployment in experiment 1 are discussed and presented.

**Fig 2:**
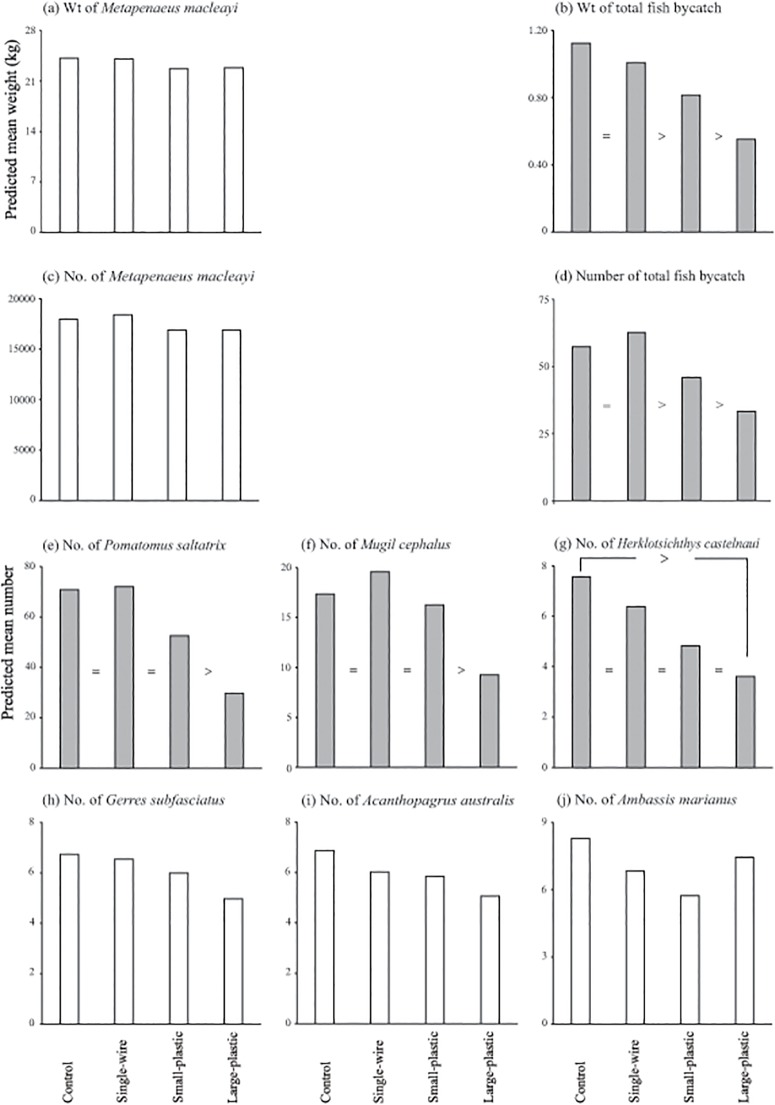
Differences in predicted mean weights of (a) school prawns, *Metapenaeus macleayi*, and (b) total fish bycatch, and the predicted mean numbers of (c) school prawns, (d) total fish bycatch, (e) tailor, *Pomatomus saltatrix*, (f) bully mullet, *Mugil cephalus*, (g) southern herring, *Herklotsichthys castelnaui*, (h) silver biddy, *Gerres subfasciatus*, (i) yellowfin bream, *Acanthopagrus australis* and (j) Ramsey’s perchlet, *Ambassis marianus* per 40-min deployment between the control and three SAFEs (single-wire, small-plastic and large-plastic) tested in experiment 1. Shaded histograms indicate significant wald *F*-values, while ‘>‘ and ‘ = ‘ indicate differences detected in false-discovery-rate pair-wise comparisons (*p*<0.05).

The anterior-trawl configuration had no significant effects on the catches, nor mean CL of *M*. *macleayi* (13.86–14.26 mm; LMM, *p*>0.05), but did significantly influence the number and weight of total fish bycatch, and the numbers of *M*. *cephalus*, *H*. *castelnaui* and *P*. *saltatrix* (LMM, *p*<0.01; Table 2; Fig 2a–2g), but not the mean size of the latter (LMM, *p*>0.05; Table 2). The significant effects on bycatch broadly were positively correlated with SAFE surface area (Fig 2b, 2d and 2e–2g). Specifically, compared to the control and the single-wire SAFE, both the small- and large-plastic SAFEs significantly and incrementally reduced the weights (by up to 27 and 51%) and numbers (by up to 26 and 47%) of total fish bycatch (FDR, *p*<0.05; Table 2, Fig 2b and 2d). The beam trawl with the large-plastic SAFE also caught significantly fewer *P*. *saltatrix* and *M*. *cephalus* than all other configurations (by up to 43 and 58%) and *H*. *castelnaui* than the control (by 49%; FDR, *p*<0.05; Table 2, Fig 2e–2g). No other fish were significantly affected by the SAFEs, although the numbers of *G*. *subfasciatus* and *A*. *australis* followed similar trends as above (LMM, *p*>0.05; Table 2, Fig 2h and 2i).

### Experiment 2: testing a SAFE on an otter trawl

The parsimonious LMM describing drag included SOG and anterior-trawl configuration as main effects, with the latter not significantly different between the control (259.5 ± 5.0 kg) and SAFE (259.7 ± 5.0 kg) trawls (*p*>0.05; Table 2). Irrespective of anterior-trawl configuration, SOG was positively associated with drag (LMM, *p*<0.05).

There was a significant difference in wing-end spreads between configurations, with the control (4.31 ± 0.21 m) spread 0.21 ± 0.05 m wider than the SAFE (LMM, *p*<0.05; Table 2). Both configurations shared a common negative association with STW (LMM, *p*<0.01). The control trawl fished a significantly greater area than the SAFE (1.43 ± 0.10 vs. 1.34 ± 0.10 ha) (LMM, *p*<0.001; Table 2).

**Fig 3:**
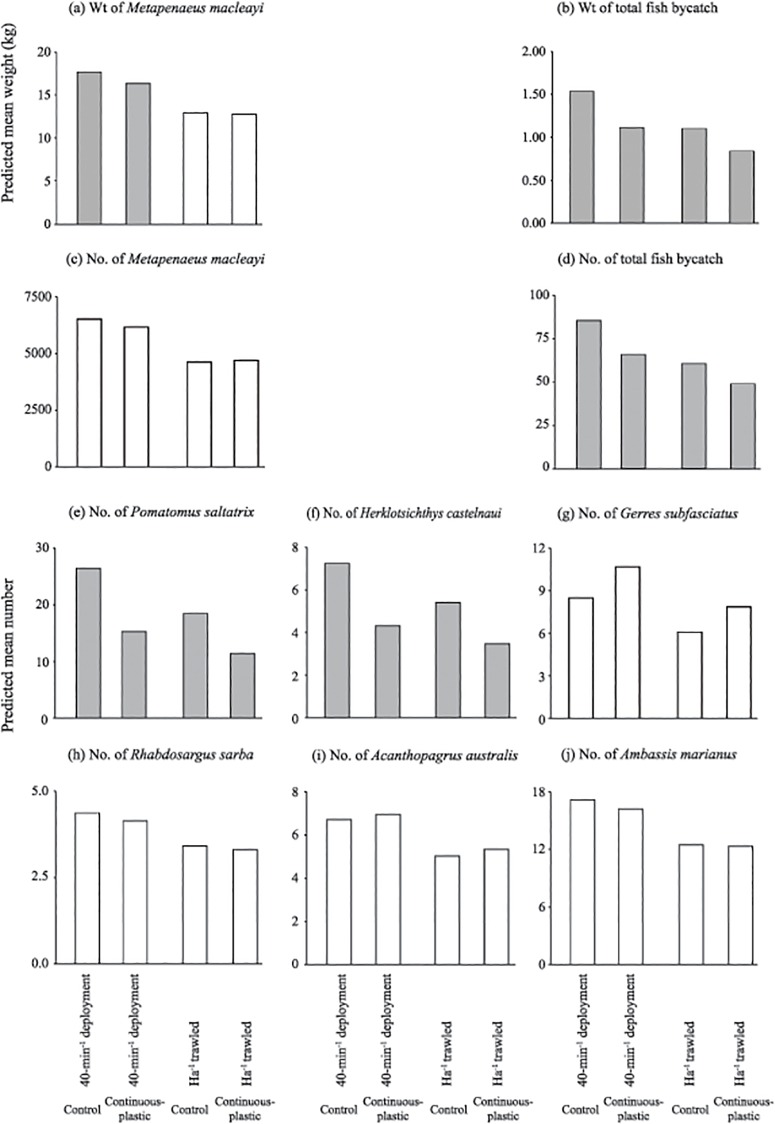
Differences in predicted mean weights of (a) school prawns, *Metapenaeus macleayi*, and (b) total fish bycatch, and the predicted mean numbers of (c) school prawns, (d) total fish bycatch, (e) tailor, *Pomatomus saltatrix*, (f) southern herring, *Herklotsichthys castelnaui*, (g) silver biddy, *Gerres subfasciatus*, (h) tarwhine, *Rhabdosargus sarba*, (i) yellowfin bream, *Acanthopagrus australis* and (j) Ramsey’s perchlet, *Ambassis marianus* per 40-min deployment and standardised to per ha trawled using the footrope contact (average wing-end spread × distance trawled) between the control otter trawl and that containing the continuous-plastic SAFE tested in experiment 2. Shaded histograms indicate significant differences detected by Wald *F*-values (*p*<0.05).

The slightly narrower trawl wing-end spread due to the continuous-plastic SAFE was reflected in a significant reduction (~7%) in the weight of *M*. *macleayi* per 40-min deployment (LMM, *p*<0.05; Table 2, Fig 3a). However, when standardised to per ha, the number and weight of *M*. *macleayi* were not significantly different between trawls (LMM, *p*>0.05; Table 2, Fig 3a and 3c), although the predicted mean CL was significantly smaller in the trawls with the SAFE (14.72 ± 0.20 mm) than the control (14.91 ± 0.20 mm) (LMM, *p*<0.05*;* Table 2).

Compared to the control, the trawl with the continuous-plastic SAFE caught significantly less total bycatch by weight (by 28%) and number (24%) and fewer *P*. *saltatrix* and *H*. *castelnaui* per 40-min deployment and ha trawled (both by up to 42%) (LMM, *p*<0.05; Table 2, Fig 3b and 3d–3f). Catches of the remaining key species were not significantly affected by the continuous-plastic SAFE (LMM, *p*>0.05; Fig 3g–3j).

## Discussion

This study validates the concept of locating simple BRDs anterior to penaeid trawls for improving their species selectivity [13, 14]. Like in our earlier, preliminary study [14], the SAFEs tested here maintained target catches at acceptable limits and, for the otter trawl, the bycatch reductions rivalled those observed for other traditional posteriorly located BRDs [6]. The SAFEs’ effectiveness can be discussed firstly according to the utility of the experimental approach, and then the related probable species-specific responses.

The limits/range of the original SAFE concept described by McHugh et al. [14] were somewhat defined in experiment 1 by incrementally testing larger modifications, involving a horizontal wire with and without small and large plastic attachments, across the beam trawl. Specifically, the sizes of the individual plastic strips—∽0.23 m long (PP strip and swivel)—were close to what we considered the maximum in terms of not contacting the top of the beam (0.76 m high), each other, nor the substrate during fishing, and potentially impacting on *M*. *macleayi* catches. However, notwithstanding the considerable bycatch reduction (up to 51%), the maintenance of *M*. *macleayi* catches at the same levels as the control, suggest that a slightly larger SAFE might have had some utility. Following this logic, we increased the area (from 11 to 23% of the trawl mouth) in the SAFE used on the otter trawl. Further, because the independent plastic strips comprising the SAFEs used on the beam trawl would have been easily entangled among the otter-trawl components (e.g. otter boards and sweep wires as they came together at the surface after each deployment), we chose a continuous-plastic strip.

While the continuous-plastic SAFE did not affect otter-trawl drag, it significantly decreased wing-end spread, the area trawled per deployment, and therefore the catches of *M*. *macleayi*. The narrower wing-end spread can probably be explained by the drag from the SAFE pulling the otter boards together which would have concomitantly reduced the drag of the trawl and ground-gear [20], providing the observed lack of change in total system resistance. It is also clear that a lower otter-board angle of attack (AOA) would have reduced the effective substrate contact and while speculative, this may have contributed to the negative impacts on *M*. *macleayi* catches—owing to fewer individuals (potentially those that were larger given the differences in mean size) being disturbed and directed into the path of the trawl [21]. Nevertheless, such catch effects were minimal and could be simply remedied by slightly increasing otter-board surface area.

The differences in wing-end spread due to the continuous-plastic SAFE had no negative effect on fish exclusion, with consistent, significant reductions both per 40 min and ha trawled. The SAFEs also maintained fish reductions between experiments, although the large-plastic SAFE used on the beam was considerably more effective (reducing total bycatch by up to 51% compared to the control) than the continuous-plastic SAFE used on the otter trawl. Although speculative, these results might be explained by the importance of visual cues in affecting fish reactions to towed gears, associated variation in trawl dynamics and potentially other environmental factors [22, 23].

Typically, the trawl capture process depends on fish being herded between the otter boards, sweep wires and trawl wings and then when fatigued, falling back into the codend [24]. This process is strongly affected by the elicited visual cues, whereby as water clarity decreases (e.g. low light or turbid conditions) so too does a fish’s ability to detect gear-components and instigate an escape response [9, 15, 22, 23, 25]. Considering the above, in experiment 1 the horizontal wires on the beam remained taut and the plastic strips probably rotated freely and individually, potentially creating a strong visual stimulus for some fish. By comparison, in experiment 2, the continuous-plastic SAFE should have provided less movement and possibly reduced stimulus. Equally important, owing to the shallow concave shape of the SAFE, the angle at the otter boards would have increased, potentially herding some fish in towards the trawl path and negating some of the effectiveness.

Beyond the specific SAFE design, we also suggest that differences in fish density and water clarity may have been important factors contributing towards the observed inter-experimental variation in performances [15, 25]. For example, all three species affected by the SAFEs, but especially *P*. *saltatrix*, were caught in large numbers (comprising 73 and 32% of the total catches in each experiment). Potentially, intra-specific reactions within schools contributed towards their escape [25]. Future research to refine the SAFE would benefit from assessing the relationship between water clarity and effectiveness. However, because the extremely poor water clarity precludes using cameras, such work will require a manipulative-type experimental approach.

While turbidity was not measured, it was assumed to be comparable between experiments based on the trawling intensity occurring in the area at the time. Available meteorological data (www.bom.gov.au) suggest ambient light may have been lower during experiment 2 with three (of five) days having greater than 50% cloud cover compared to three (of seven) in experiment 1. The selectivity of *H*. *castelnaui* could have been influenced by the lower ambient light level, which limits the ability of some species to detect trawls [26].

Irrespective of the variability among performances, the observed bycatch reductions, combined with the simplicity and low cost of a SAFE (which should promote adoption as part of a legislated suite of existing, but more complex BRD designs in this fishery) support ongoing testing and refinement. As part of such work, it would be worthwhile to explore ways in which SAFEs could be engineered to concomitantly improve system engineering (and therefore reduce fuel usage). One potential option might be to use the SAFE to more accurately regulate otter-board AOA. It is well established that otter boards represent a large proportion (up to ∽30%) of trawl-system drag, which directly correlates to their AOA [27]. Most designs have a high AOA (>30°) to increase stability during deployment, but can have greater operational efficiency at AOAs as low as 20° [27]. Locating an appropriate length of SAFE at the leading edge of otter boards might achieve a lower AOA, and if so reduce some unnecessary system drag. Given the high global price of fuel, even a slight reduction in drag would help to promote industry adoption of the SAFE concept.

Another modification to improve the utility of the SAFE would be to configure a design that maintains a convex shape (away from the trawl mouth); potentially, helping to disperse fish away from the trawl [13]. While this may be difficult to achieve on an otter trawl (due to configuration constraints) such a design might be applicable on a beam trawl, and warrants further testing.

It is clear that trawl gear has evolved to exploit the behavioural and physiological responses of targeted species, but often with concomitant negative impacts on unwanted catches. Retrospectively fitted BRDs have been, and will continue to remain, an important applied strategy for mitigating bycatches, and ideally their associated unaccounted fishing mortality. Based on the results here, the SAFE concept might represent an effective approach for improving the selectivity of penaeid trawls.

## Supporting Information

S1 Table Operational data, from sensors (load cells, and GPS), and catch statistics from experiment 1—testing three different SAFEs on a beam trawl. (DOCX)

S2 Table Operational data, from sensors (load cells, GPS, and NOTUS), and catch statistics from experiment 2—testing a SAFE on an otter trawl. (DOCX)
