# Supplementary material for: A ‘Simple Anterior Fish Excluder’ (SAFE) for Mitigating Penaeid-Trawl Bycatch
Source: PLoS One. 2015 Apr 2;10(4):e0123124. doi: 10.1371/journal.pone.0123124 (PMC4383628; doi:10.1371/journal.pone.0123124)
Supplement: S1 Table — (DOCX) [file pone.0123124.s001.docx]

Table S1. Operational data, from sensors (load cells, and GPS), and catch statistics from experiment 1— testing three different SAFEs on a beam trawl.

|  |  |  |  |  |  |  |  |  |  |  |  |  |  |  |  |  |  |  |  |  |  |  |  |  |  |  |  |
| --- | --- | --- | --- | --- | --- | --- | --- | --- | --- | --- | --- | --- | --- | --- | --- | --- | --- | --- | --- | --- | --- | --- | --- | --- | --- | --- | --- |
| **Day no** | **Haul** | **Treatment** | **Wing-end spread** | **Trawl load (kg)** | **Distance trawled (m)** | **Wing-end area trawled ( Ha)** | **Average speed (m/sec)** | **Paired fuel used (L)** | **Total catch Weight (kg) 40-min** | **Total catch weight (kg) Ha^-1^** | **Weight School prawns (kg) 40-min** | **Weight School prawns (kg) Ha^-1^** | **No. School prawns 40-min** | **No. School prawns Ha^-1^** | **Cl School prawns** | **Number** | | | | | | | | | | | |
|  |  |  |  |  |  |  |  |  |  |  |  |  |  |  |  | **Yellowfin bream 40-min** | **Yellowfin bream Ha^-1^** | **Bully mullet 40-min** | **Bully mullet Ha^-1^** | **Ramsey's perchlet 40-min** | **Ramsey's perchlet Ha^-1^** | **Southern herring 40-min** | **Southern herring Ha^-1^** | **Silver biddy 40-min** | **Silver biddy Ha^-1^** | **Tailor 40-min** | **Tailor Ha^-1^** |
| 1 | 2 | Control | 6 | 207.32 | 3.28 | 1.97 | 1.37 | 7.2 | 17.2 | 8.75 | 14.00 | 7.12 | 7.91 | 4.02 | 13.00 | 2 | 1.02 | 52 | 26.44 | 2 | 1.02 | 0 | 0.00 | 2 | 1.02 | 152 | 77.28 |
| 1 | 3 | Control | 6 | 216.46 | 3.32 | 1.99 | 1.38 | 7.4 | 13.2 | 6.64 | 9.20 | 4.63 | 4.51 | 2.27 | 14.50 | 8 | 4.02 | 1 | 0.50 | 9 | 4.52 | 1 | 0.50 | 2 | 1.01 | 48 | 24.13 |
| 1 | 6 | Control | 6 | 208.64 | 3.30 | 1.98 | 1.37 | 7.2 | 15.8 | 7.99 | 14.00 | 7.08 | 6.63 | 3.35 | 14.00 | 8 | 4.04 | 0 | 0.00 | 4 | 2.02 | 5 | 2.53 | 1 | 0.51 | 4 | 2.02 |
| 2 | 1 | Control | 6 | 206.67 | 3.32 | 1.99 | 1.38 | 6.7 | 29.48 | 14.82 | 26.50 | 13.32 | 12.71 | 6.39 | 14.50 | 8 | 4.02 | 12 | 6.03 | 10 | 5.03 | 32 | 16.09 | 6 | 3.02 | 116 | 58.32 |
| 2 | 4 | Control | 6 | 203.12 | 3.48 | 2.09 | 1.45 | 6.7 | 2.55 | 1.22 | 0.35 | 0.17 | 0.15 | 0.07 | 14.00 | 8 | 3.83 | 3 | 1.44 | 0 | 0.00 | 21 | 10.05 | 9 | 4.31 | 46 | 22.02 |
| 2 | 6 | Control | 6 | 229.96 | 3.20 | 1.92 | 1.33 | 7 | 2.42 | 1.26 | 0.40 | 0.21 | 0.19 | 0.10 | 14.80 | 2 | 1.04 | 1 | 0.52 | 5 | 2.60 | 11 | 5.72 | 5 | 2.60 | 43 | 22.37 |
| 3 | 1 | Control | 6 | 204.88 | 3.19 | 1.91 | 1.33 | 6.7 | 18.86 | 9.87 | 16.10 | 8.42 | 6.93 | 3.63 | 13.50 | 2 | 1.05 | 48 | 25.11 | 4 | 2.09 | 6 | 3.14 | 2 | 1.05 | 164 | 85.81 |
| 3 | 2 | Control | 6 | 213.09 | 3.20 | 1.92 | 1.33 | 6.9 | 23.98 | 12.47 | 21.90 | 11.39 | 10.10 | 5.26 | 15.00 | 4 | 2.08 | 49 | 25.49 | 1 | 0.52 | 0 | 0.00 | 0 | 0.00 | 66 | 34.33 |
| 3 | 5 | Control | 6 | 212.02 | 3.30 | 1.98 | 1.37 | 7.2 | 23.6 | 11.93 | 21.20 | 10.72 | 9.13 | 4.61 | 14.09 | 4 | 2.02 | 40 | 20.22 | 4 | 2.02 | 2 | 1.01 | 0 | 0.00 | 138 | 69.77 |
| 4 | 2 | Control | 6 | 209.92 | 3.28 | 1.97 | 1.37 | 7 | 10.5 | 5.34 | 8.40 | 4.27 | 3.42 | 1.74 | 15.00 | 1 | 0.51 | 7 | 3.56 | 5 | 2.54 | 0 | 0.00 | 0 | 0.00 | 46 | 23.39 |
| 4 | 4 | Control | 6 | 208.89 | 3.15 | 1.89 | 1.31 | 6.8 | 24.02 | 12.72 | 22.00 | 11.65 | 8.38 | 4.44 | 15.00 | 0 | 0.00 | 2 | 1.06 | 2 | 1.06 | 0 | 0.00 | 0 | 0.00 | 27 | 14.29 |
| 4 | 5 | Control | 6 | 208.88 | 3.32 | 1.99 | 1.38 | 7.1 | 21.74 | 10.93 | 19.80 | 9.95 | 8.77 | 4.41 | 13.92 | 0 | 0.00 | 15 | 7.54 | 1 | 0.50 | 1 | 0.50 | 2 | 1.01 | 64 | 32.18 |
| 5 | 2 | Control | 6 | 194.37 | 3.19 | 1.91 | 1.33 | 6.7 | 25.22 | 13.20 | 23.00 | 12.03 | 9.24 | 4.83 | 14.08 | 2 | 1.05 | 18 | 9.42 | 4 | 2.09 | 4 | 2.09 | 0 | 0.00 | 124 | 64.88 |
| 5 | 4 | Control | 6 | 212.28 | 3.22 | 1.93 | 1.34 | 7 | 40.04 | 20.71 | 39.00 | 20.17 | 17.95 | 9.29 | 13.58 | 2 | 1.03 | 6 | 3.10 | 4 | 2.07 | 3 | 1.55 | 0 | 0.00 | 57 | 29.48 |
| 5 | 5 | Control | 6 | 217.11 | 3.20 | 1.92 | 1.33 | 7 | 64.32 | 33.46 | 62.60 | 32.56 | 27.72 | 14.42 | 12.50 | 1 | 0.52 | 12 | 6.24 | 5 | 2.60 | 4 | 2.08 | 1 | 0.52 | 53 | 27.57 |
| 6 | 1 | Control | 6 | 205.75 | 3.15 | 1.89 | 1.31 | 6.9 | 3.2 | 1.69 | 2.32 | 1.23 | 0.85 | 0.45 | 15.50 | 0 | 0.00 | 16 | 8.47 | 2 | 1.06 | 16 | 8.47 | 2 | 1.06 | 32 | 16.94 |
| 6 | 3 | Control | 6 | No Data | 3.09 | 1.86 | 1.29 | 6.8 | 12.08 | 6.51 | 10.30 | 5.55 | 4.10 | 2.21 | 13.43 | 0 | 0.00 | 13 | 7.01 | 3 | 1.62 | 2 | 1.08 | 2 | 1.08 | 38 | 20.48 |
| 6 | 6 | Control | 6 | 212.35 | 3.26 | 1.96 | 1.36 | 7.1 | 10.6 | 5.42 | 8.20 | 4.19 | 3.58 | 1.83 | 14.00 | 2 | 1.02 | 16 | 8.18 | 8 | 4.09 | 10 | 5.11 | 0 | 0.00 | 48 | 24.54 |
| 7 | 1 | Control | 6 | 206.61 | 3.61 | 2.17 | 1.50 | 6.1 | 14.52 | 6.70 | 10.60 | 4.89 | 3.99 | 1.84 | 15.00 | 24 | 11.08 | 12 | 5.54 | 20 | 9.23 | 12 | 5.54 | 66 | 30.46 | 22 | 10.15 |
| 7 | 2 | Control | 6 | 206.59 | 2.80 | 1.68 | 1.17 | 6.3 | 9.92 | 5.91 | 8.50 | 5.07 | 3.34 | 1.99 | 14.80 | 5 | 2.98 | 3 | 1.79 | 20 | 11.92 | 8 | 4.77 | 14 | 8.34 | 13 | 7.75 |
| 7 | 5 | Control | 6 | 196.97 | 3.50 | 2.10 | 1.46 | 6.2 | 9.2 | 4.38 | 4.10 | 1.95 | 1.81 | 0.86 | 15.00 | 18 | 8.57 | 15 | 7.14 | 15 | 7.14 | 9 | 4.29 | 21 | 10.00 | 57 | 27.14 |
| 1 | 4 | Wire | 6 | 207.53 | 3.09 | 1.86 | 1.29 | 7.1 | 19.92 | 10.73 | 18.20 | 9.81 | 9.86 | 5.31 | 13.42 | 14 | 7.54 | 10 | 5.39 | 16 | 8.62 | 4 | 2.16 | 2 | 1.08 | 48 | 25.87 |
| 1 | 2 | Wire | 6 | 187.79 | 3.28 | 1.97 | 1.37 | 7.2 | 17.4 | 8.85 | 13.50 | 6.86 | 0.00 | 0.00 | No Data | 4 | 2.03 | 40 | 20.34 | 2 | 1.02 | 12 | 6.10 | 0 | 0.00 | 178 | 90.50 |
| 1 | 5 | Wire | 6 | 201.09 | 3.19 | 1.91 | 1.33 | 7.4 | 18.6 | 9.73 | 15.80 | 8.27 | 7.45 | 3.90 | 13.50 | 3 | 1.57 | 0 | 0.00 | 7 | 3.66 | 1 | 0.52 | 5 | 2.62 | 67 | 35.06 |
| 2 | 2 | Wire | 6 | 169.57 | 3.20 | 1.92 | 1.33 | 6.5 | 15.88 | 8.26 | 13.10 | 6.81 | 6.22 | 3.23 | 13.75 | 6 | 3.12 | 6 | 3.12 | 5 | 2.60 | 20 | 10.40 | 3 | 1.56 | 81 | 42.14 |
| 2 | 5 | Wire | 6 | 242.58 | 3.32 | 1.99 | 1.38 | 7.5 | 2.62 | 1.32 | 0.00 | 0.00 | 0.00 | 0.00 | 0.00 | 2 | 1.01 | 0 | 0.00 | 0 | 0.00 | 19 | 9.55 | 4 | 2.01 | 6 | 3.02 |
| 2 | 6 | Wire | 6 | 218.55 | 3.20 | 1.92 | 1.33 | 7 | 3.54 | 1.84 | 0.34 | 0.18 | 0.16 | 0.08 | 14.50 | 2 | 1.04 | 1 | 0.52 | 3 | 1.56 | 18 | 9.36 | 4 | 2.08 | 41 | 21.33 |
| 3 | 2 | Wire | 6 | 191.46 | 3.20 | 1.92 | 1.33 | 6.9 | 24.8 | 12.90 | 22.50 | 11.70 | 9.49 | 4.94 | 14.08 | 0 | 0.00 | 72 | 37.45 | 0 | 0.00 | 0 | 0.00 | 0 | 0.00 | 100 | 52.02 |
| 3 | 3 | Wire | 6 | 201.99 | 3.20 | 1.92 | 1.33 | 6.7 | 25.22 | 13.12 | 23.20 | 12.07 | 11.01 | 5.73 | 13.50 | 2 | 1.04 | 41 | 21.33 | 0 | 0.00 | 0 | 0.00 | 0 | 0.00 | 84 | 43.70 |
| 3 | 4 | Wire | 6 | 207.24 | 3.28 | 1.97 | 1.37 | 7 | 24.62 | 12.52 | 21.70 | 11.03 | 10.20 | 5.19 | 13.50 | 0 | 0.00 | 29 | 14.74 | 1 | 0.51 | 1 | 0.51 | 0 | 0.00 | 80 | 40.67 |
| 4 | 1 | Wire | 6 | 201.04 | 3.09 | 1.86 | 1.29 | 6.6 | 23.26 | 12.53 | 21.20 | 11.42 | 10.30 | 5.55 | 13.50 | 1 | 0.54 | 3 | 1.62 | 12 | 6.47 | 0 | 0.00 | 0 | 0.00 | 40 | 21.56 |
| 4 | 4 | Wire | 6 | 197.89 | 3.15 | 1.89 | 1.31 | 6.8 | 19.78 | 10.47 | 18.10 | 9.58 | 7.54 | 3.99 | 14.00 | 0 | 0.00 | 21 | 11.12 | 0 | 0.00 | 0 | 0.00 | 0 | 0.00 | 23 | 12.18 |
| 4 | 6 | Wire | 6 | 210.61 | 3.22 | 1.93 | 1.34 | 7 | 17 | 8.79 | 16.40 | 8.48 | 7.43 | 3.84 | 13.58 | 4 | 2.07 | 1 | 0.52 | 2 | 1.03 | 0 | 0.00 | 2 | 1.03 | 9 | 4.65 |
| 5 | 1 | Wire | 6 | 196.12 | 3.13 | 1.88 | 1.30 | 6.6 | 15.94 | 8.49 | 14.06 | 7.49 | 5.45 | 2.90 | 15.00 | 0 | 0.00 | 2 | 1.07 | 2 | 1.07 | 2 | 1.07 | 2 | 1.07 | 114 | 60.71 |
| 5 | 2 | Wire | 6 | 198.42 | 3.19 | 1.91 | 1.33 | 6.7 | 24.34 | 12.74 | 22.20 | 11.62 | 7.94 | 4.16 | 15.07 | 0 | 0.00 | 20 | 10.46 | 20 | 10.46 | 2 | 1.05 | 0 | 0.00 | 84 | 43.95 |
| 5 | 6 | Wire | 6 | 229.90 | 3.33 | 2.00 | 1.39 | 7.6 | 34.56 | 17.28 | 32.30 | 16.15 | 12.84 | 6.42 | 13.50 | 1 | 0.50 | 5 | 2.50 | 5 | 2.50 | 7 | 3.50 | 1 | 0.50 | 54 | 27.00 |
| 6 | 1 | Wire | 6 | 214.19 | 3.15 | 1.89 | 1.31 | 6.9 | 4.4 | 2.33 | 2.10 | 1.11 | 0.77 | 0.41 | 14.00 | 1 | 0.53 | 26 | 13.76 | 3 | 1.59 | 7 | 3.71 | 1 | 0.53 | 57 | 30.17 |
| 6 | 4 | Wire | 6 | 207.39 | 3.17 | 1.90 | 1.32 | 6.9 | 14.7 | 7.74 | 12.60 | 6.63 | 4.92 | 2.59 | 14.00 | 2 | 1.05 | 33 | 17.37 | 3 | 1.58 | 0 | 0.00 | 0 | 0.00 | 39 | 20.52 |
| 6 | 5 | Wire | 6 | 217.40 | 3.22 | 1.93 | 1.34 | 7 | 10.54 | 5.45 | 9.10 | 4.71 | 3.47 | 1.79 | 14.13 | 1 | 0.52 | 13 | 6.72 | 2 | 1.03 | 7 | 3.62 | 1 | 0.52 | 60 | 31.03 |
| 7 | 2 | Wire | 6 | 199.97 | 2.80 | 1.68 | 1.17 | 6.3 | 11.66 | 6.95 | 9.50 | 5.66 | 3.61 | 2.15 | 14.58 | 9 | 5.36 | 4 | 2.38 | 16 | 9.54 | 7 | 4.17 | 20 | 11.92 | 19 | 11.32 |
| 7 | 3 | Wire | 6 | 192.93 | 3.07 | 1.84 | 1.28 | 6.3 | 13.16 | 7.13 | 10.00 | 5.42 | 3.96 | 2.15 | 14.50 | 34 | 18.43 | 12 | 6.51 | 8 | 4.34 | 2 | 1.08 | 66 | 35.78 | 36 | 19.52 |
| 7 | 4 | Wire | 6 | 221.92 | 3.04 | 1.82 | 1.27 | 6.4 | 11.92 | 6.54 | 9.80 | 5.38 | 4.33 | 2.38 | 14.57 | 44 | 24.14 | 0 | 0.00 | 20 | 10.97 | 2 | 1.10 | 42 | 23.05 | 24 | 13.17 |
| 1 | 1 | Small plastic | 6 | 185.59 | 3.22 | 1.93 | 1.34 | 6.7 | 13.03 | 6.74 | 11.00 | 5.69 | 5.00 | 2.59 | 14.00 | 21 | 10.86 | 8 | 4.14 | 8 | 4.14 | 8 | 4.14 | 6 | 3.10 | 75 | 38.79 |
| 1 | 5 | Small plastic | 6 | 219.07 | 3.19 | 1.91 | 1.33 | 7.4 | 20.19 | 10.56 | 18.00 | 9.42 | 3.20 | 1.67 | 14.50 | 5 | 2.62 | 0 | 0.00 | 11 | 5.76 | 0 | 0.00 | 1 | 0.52 | 88 | 46.04 |
| 1 | 3 | Small plastic | 6 | 202.76 | 3.32 | 1.99 | 1.38 | 7.4 | 10.3 | 5.18 | 6.50 | 3.27 | 9.34 | 4.70 | 13.15 | 16 | 8.04 | 7 | 3.52 | 1 | 0.50 | 2 | 1.01 | 1 | 0.50 | 75 | 37.71 |
| 2 | 1 | Small plastic | 6 | 207.00 | 3.32 | 1.99 | 1.38 | 6.7 | 31.94 | 16.06 | 29.20 | 14.68 | 14.01 | 7.04 | 13.50 | 3 | 1.51 | 11 | 5.53 | 3 | 1.51 | 2 | 1.01 | 6 | 3.02 | 46 | 23.13 |
| 2 | 3 | Small plastic | 6 | 218.87 | 3.32 | 1.99 | 1.38 | 7.1 | 44.8 | 22.52 | 43.00 | 21.62 | 21.09 | 10.60 | 13.00 | 1 | 0.50 | 9 | 4.52 | 3 | 1.51 | 1 | 0.50 | 2 | 1.01 | 43 | 21.62 |
| 2 | 5 | Small plastic | 6 | 225.63 | 3.32 | 1.99 | 1.38 | 7.5 | 3.11 | 1.56 | 0.00 | 0.00 | 0.00 | 0.00 | 0.00 | 2 | 1.01 | 1 | 0.50 | 0 | 0.00 | 6 | 3.02 | 18 | 9.05 | 0 | 0.00 |
| 3 | 3 | Small plastic | 6 | 203.32 | 3.20 | 1.92 | 1.33 | 6.7 | 24.7 | 12.85 | 22.50 | 11.70 | 10.47 | 5.45 | 14.50 | 1 | 0.52 | 16 | 8.32 | 2 | 1.04 | 2 | 1.04 | 0 | 0.00 | 77 | 40.05 |
| 3 | 5 | Small plastic | 6 | 222.28 | 3.30 | 1.98 | 1.37 | 7.2 | 24.1 | 12.18 | 21.50 | 10.87 | 10.38 | 5.25 | 13.00 | 0 | 0.00 | 19 | 9.61 | 2 | 1.01 | 1 | 0.51 | 0 | 0.00 | 77 | 38.93 |
| 3 | 6 | Small plastic | 6 | 214.57 | 3.33 | 2.00 | 1.39 | 7.1 | 8.08 | 4.04 | 6.90 | 3.45 | 3.17 | 1.59 | 13.92 | 1 | 0.50 | 7 | 3.50 | 2 | 1.00 | 5 | 2.50 | 0 | 0.00 | 40 | 20.00 |
| 4 | 2 | Small plastic | 6 | 206.40 | 3.28 | 1.97 | 1.37 | 7 | 12.24 | 6.22 | 9.20 | 4.68 | 4.12 | 2.10 | 13.00 | 5 | 2.54 | 10 | 5.08 | 1 | 0.51 | 0 | 0.00 | 1 | 0.51 | 41 | 20.85 |
| 4 | 3 | Small plastic | 6 | 208.51 | 3.20 | 1.92 | 1.33 | 6.8 | 16.06 | 8.35 | 14.80 | 7.70 | 6.02 | 3.13 | 15.00 | 0 | 0.00 | 20 | 10.40 | 0 | 0.00 | 0 | 0.00 | 0 | 0.00 | 20 | 10.40 |
| 4 | 6 | Small plastic | 6 | 211.92 | 3.22 | 1.93 | 1.34 | 7 | 14.88 | 7.70 | 14.20 | 7.34 | 6.14 | 3.18 | 14.00 | 1 | 0.52 | 0 | 0.00 | 5 | 2.59 | 0 | 0.00 | 1 | 0.52 | 7 | 3.62 |
| 5 | 1 | Small plastic | 6 | 192.52 | 3.13 | 1.88 | 1.30 | 6.6 | 14.28 | 7.60 | 11.80 | 6.28 | 4.61 | 2.46 | 14.00 | 4 | 2.13 | 10 | 5.33 | 2 | 1.07 | 0 | 0.00 | 4 | 2.13 | 82 | 43.67 |
| 5 | 3 | Small plastic | 6 | 209.74 | 3.24 | 1.94 | 1.35 | 6.8 | 38.06 | 19.57 | 37.00 | 19.03 | 13.56 | 6.97 | 14.00 | 0 | 0.00 | 5 | 2.57 | 1 | 0.51 | 0 | 0.00 | 0 | 0.00 | 50 | 25.71 |
| 5 | 5 | Small plastic | 6 | 204.41 | 3.20 | 1.92 | 1.33 | 7 | 61.12 | 31.79 | 60.10 | 31.26 | 24.62 | 12.81 | 13.64 | 0 | 0.00 | 2 | 1.04 | 0 | 0.00 | 9 | 4.68 | 1 | 0.52 | 36 | 18.73 |
| 6 | 2 | Small plastic | 6 | 173.32 | 3.22 | 1.93 | 1.34 | 7.2 | 9.46 | 4.89 | 8.40 | 4.34 | 2.98 | 1.54 | 14.50 | 0 | 0.00 | 17 | 8.79 | 1 | 0.52 | 2 | 1.03 | 0 | 0.00 | 31 | 16.03 |
| 6 | 4 | Small plastic | 6 | 206.23 | 3.17 | 1.90 | 1.32 | 6.9 | 16.62 | 8.75 | 14.20 | 7.47 | 4.91 | 2.58 | 14.00 | 2 | 1.05 | 18 | 9.47 | 7 | 3.68 | 0 | 0.00 | 0 | 0.00 | 39 | 20.52 |
| 6 | 6 | Small plastic | 6 | 206.78 | 3.26 | 1.96 | 1.36 | 7.1 | 8.28 | 4.23 | 7.20 | 3.68 | 2.94 | 1.50 | 12.50 | 0 | 0.00 | 29 | 14.83 | 5 | 2.56 | 2 | 1.02 | 0 | 0.00 | 45 | 23.01 |
| 7 | 1 | Small plastic | 6 | 222.05 | 3.61 | 2.17 | 1.50 | 6.1 | 13.6 | 6.28 | 9.50 | 4.38 | 3.70 | 1.71 | 15.00 | 33 | 15.23 | 18 | 8.31 | 21 | 9.69 | 57 | 26.31 | 57 | 26.31 | 45 | 20.77 |
| 7 | 3 | Small plastic | 6 | 206.94 | 3.07 | 1.84 | 1.28 | 6.3 | 11.2 | 6.07 | 8.40 | 4.55 | 3.27 | 1.77 | 13.92 | 26 | 14.10 | 5 | 2.71 | 10 | 5.42 | 2 | 1.08 | 39 | 21.14 | 20 | 10.84 |
| 7 | 6 | Small plastic | 6 | 217.77 | 3.15 | 1.89 | 1.31 | 6.3 | 5.68 | 3.01 | 2.40 | 1.27 | 0.98 | 0.52 | 14.00 | 3 | 1.59 | 10 | 5.29 | 10 | 5.29 | 2 | 1.06 | 3 | 1.59 | 37 | 19.59 |
| 1 | 6 | Large plastic | 6 | 183.23 | 3.30 | 1.98 | 1.37 | 7.2 | 16.12 | 8.15 | 14.50 | 7.33 | 6.78 | 3.43 | 15.00 | 7 | 3.54 | 0 | 0.00 | 15 | 7.58 | 4 | 2.02 | 5 | 2.53 | 2 | 1.01 |
| 1 | 1 | Large plastic | 6 | 205.67 | 3.22 | 1.93 | 1.34 | 6.7 | 13.07 | 6.76 | 11.50 | 5.95 | 4.92 | 2.54 | 14.00 | 9 | 4.65 | 3 | 1.55 | 4 | 2.07 | 1 | 0.52 | 7 | 3.62 | 40 | 20.69 |
| 1 | 4 | Large plastic | 6 | 212.68 | 3.09 | 1.86 | 1.29 | 7.1 | 15.8 | 8.51 | 14.00 | 7.54 | 6.48 | 3.49 | 13.50 | 5 | 2.69 | 0 | 0.00 | 5 | 2.69 | 5 | 2.69 | 0 | 0.00 | 56 | 30.18 |
| 2 | 2 | Large plastic | 6 | 207.57 | 3.20 | 1.92 | 1.33 | 6.5 | 13.96 | 7.26 | 11.20 | 5.83 | 5.61 | 2.92 | 13.75 | 6 | 3.12 | 2 | 1.04 | 3 | 1.56 | 1 | 0.52 | 0 | 0.00 | 27 | 14.05 |
| 2 | 3 | Large plastic | 6 | 218.89 | 3.32 | 1.99 | 1.38 | 7.1 | 45.6 | 22.93 | 44.30 | 22.27 | 23.10 | 11.61 | 12.50 | 1 | 0.50 | 15 | 7.54 | 4 | 2.01 | 0 | 0.00 | 2 | 1.01 | 25 | 12.57 |
| 2 | 4 | Large plastic | 6 | 199.21 | 3.48 | 2.09 | 1.45 | 6.7 | 2.49 | 1.19 | 0.35 | 0.17 | 0.15 | 0.07 | 14.00 | 5 | 2.39 | 0 | 0.00 | 0 | 0.00 | 2 | 0.96 | 8 | 3.83 | 1 | 0.48 |
| 3 | 1 | Large plastic | 6 | 210.71 | 3.19 | 1.91 | 1.33 | 6.7 | 16.42 | 8.59 | 14.50 | 7.59 | 6.27 | 3.28 | 14.50 | 0 | 0.00 | 32 | 16.74 | 2 | 1.05 | 1 | 0.52 | 1 | 0.52 | 32 | 16.74 |
| 3 | 4 | Large plastic | 6 | 213.30 | 3.28 | 1.97 | 1.37 | 7 | 18.22 | 9.26 | 16.50 | 8.39 | 7.21 | 3.67 | 14.00 | 0 | 0.00 | 20 | 10.17 | 1 | 0.51 | 0 | 0.00 | 0 | 0.00 | 32 | 16.27 |
| 3 | 6 | Large plastic | 6 | 196.57 | 3.33 | 2.00 | 1.39 | 7.1 | 7.26 | 3.63 | 5.90 | 2.95 | 2.54 | 1.27 | 15.00 | 1 | 0.50 | 2 | 1.00 | 1 | 0.50 | 0 | 0.00 | 0 | 0.00 | 19 | 9.50 |
| 4 | 1 | Large plastic | 6 | 205.62 | 3.09 | 1.86 | 1.29 | 6.6 | 19.54 | 10.53 | 18.60 | 10.02 | 9.24 | 4.98 | 13.00 | 0 | 0.00 | 6 | 3.23 | 7 | 3.77 | 0 | 0.00 | 0 | 0.00 | 12 | 6.47 |
| 4 | 3 | Large plastic | 6 | 200.04 | 3.20 | 1.92 | 1.33 | 6.8 | 18.72 | 9.74 | 17.80 | 9.26 | 7.65 | 3.98 | 13.92 | 1 | 0.52 | 3 | 1.56 | 1 | 0.52 | 1 | 0.52 | 0 | 0.00 | 20 | 10.40 |
| 4 | 5 | Large plastic | 6 | 217.39 | 3.32 | 1.99 | 1.38 | 7.1 | 18.4 | 9.25 | 17.20 | 8.65 | 7.76 | 3.90 | 13.00 | 0 | 0.00 | 7 | 3.52 | 1 | 0.50 | 0 | 0.00 | 0 | 0.00 | 13 | 6.54 |
| 5 | 3 | Large plastic | 6 | 206.26 | 3.24 | 1.94 | 1.35 | 6.8 | 49.6 | 25.51 | 48.50 | 24.94 | 18.76 | 9.65 | 14.50 | 0 | 0.00 | 9 | 4.63 | 5 | 2.57 | 1 | 0.51 | 4 | 2.06 | 42 | 21.60 |
| 5 | 4 | Large plastic | 6 | 218.15 | 3.22 | 1.93 | 1.34 | 7 | 36.3 | 18.77 | 34.10 | 17.64 | 13.85 | 7.16 | 13.50 | 1 | 0.52 | 2 | 1.03 | 11 | 5.69 | 0 | 0.00 | 0 | 0.00 | 31 | 16.03 |
| 5 | 6 | Large plastic | 6 | 221.01 | 3.33 | 2.00 | 1.39 | 7.6 | 35.86 | 17.93 | 35.20 | 17.60 | 14.41 | 7.20 | 15.00 | 3 | 1.50 | 0 | 0.00 | 2 | 1.00 | 11 | 5.50 | 0 | 0.00 | 35 | 17.50 |
| 6 | 2 | Large plastic | 6 | 192.34 | 3.22 | 1.93 | 1.34 | 7.2 | 10.23 | 5.29 | 8.35 | 4.32 | 2.93 | 1.52 | 15.57 | 1 | 0.52 | 8 | 4.14 | 3 | 1.55 | 5 | 2.59 | 0 | 0.00 | 23 | 11.90 |
| 6 | 3 | Large plastic | 6 |  | 3.09 | 1.86 | 1.29 | 6.8 | 11.32 | 6.10 | 10.00 | 5.39 | 3.73 | 2.01 | 14.00 | 0 | 0.00 | 5 | 2.69 | 3 | 1.62 | 1 | 0.54 | 0 | 0.00 | 21 | 11.32 |
| 6 | 5 | Large plastic | 6 | 203.63 | 3.22 | 1.93 | 1.34 | 7 | 10.76 | 5.57 | 9.20 | 4.76 | 3.49 | 1.80 | 15.00 | 4 | 2.07 | 8 | 4.14 | 8 | 4.14 | 3 | 1.55 | 1 | 0.52 | 31 | 16.03 |
| 7 | 4 | Large plastic | 6 | 214.49 | 3.04 | 1.82 | 1.27 | 6.4 | 11.32 | 6.21 | 9.40 | 5.16 | 3.70 | 2.03 | 14.64 | 15 | 8.23 | 0 | 0.00 | 13 | 7.13 | 1 | 0.55 | 29 | 15.91 | 9 | 4.94 |
| 7 | 5 | Large plastic | 6 | 201.79 | 3.50 | 2.10 | 1.46 | 6.2 | 8.08 | 3.85 | 3.80 | 1.81 | 1.48 | 0.71 | 14.00 | 5 | 2.38 | 3 | 1.43 | 14 | 6.67 | 3 | 1.43 | 22 | 10.48 | 17 | 8.09 |
| 7 | 6 | Large plastic | 6 | 207.78 | 3.15 | 1.89 | 1.31 | 6.3 | 6.02 | 3.19 | 2.30 | 1.22 | 1.07 | 0.57 | 13.67 | 1 | 0.53 | 19 | 10.06 | 17 | 9.00 | 1 | 0.53 | 0 | 0.00 | 23 | 12.18 |
