# Supplementary material for: A ‘Simple Anterior Fish Excluder’ (SAFE) for Mitigating Penaeid-Trawl Bycatch
Source: PLoS One. 2015 Apr 2;10(4):e0123124. doi: 10.1371/journal.pone.0123124 (PMC4383628; doi:10.1371/journal.pone.0123124)
Supplement: S2 Table — (DOCX) [file pone.0123124.s002.docx]

Table S2. Operational data, from sensors (load cells, GPS, and NOTUS), and catch statistics from experiment 2—testing a SAFE on an otter trawl

| **Day no** | **Haul** | **Treatment** | **Wing-end spread** | **Trawl load (kg)** | **Distance trawled (km)** | **Wing-end area trawled ( Ha)** | **Average speed (m/sec)** | **Paired fuel used (L)** | **Total catch Weight 40-min** | **Total catch weight Ha^-1^** | **Weight School prawns 40-min** | **Weight School prawns Ha^-1^** | **No. School prawns 40-min** | **No. School prawns Ha^-1^** | **Cl School prawns** | **Number** | | | | | | | | | | | |
| --- | --- | --- | --- | --- | --- | --- | --- | --- | --- | --- | --- | --- | --- | --- | --- | --- | --- | --- | --- | --- | --- | --- | --- | --- | --- | --- | --- |
|  |  |  |  |  |  |  |  |  |  |  |  |  |  |  |  | **Yellowfin bream 40-min** | **Yellowfin bream Ha^-1^** | **Ramsey's perchlet 4-min** | **Ramsey's perchlet Ha^-1^** | **Southern herring 40-min** | **Southern herring Ha^-1^** | **Silver biddy 40-min** | **Silver biddy Ha^-1^** | **Tailor 40-min** | **Tailor Ha^-1^** | **Tarwhine 40-min** | **Tarwhine Ha^-1^** |
| 1 | 1 | Control | 4.28 | 251.42 | 3.20 | 1.37 | 1.33 | 9.7 | 17.50 | 12.78 | 16.80 | 12.27 | 6641.97 | 4849.24 | 15.32 | 0 | 0.00 | 4 | 2.92 | 1 | 0.73 | 0 | 0.00 | 15 | 10.95 | 0 | 0.00 |
| 1 | 2 | Control | 4.24 | 275.44 | 3.15 | 1.34 | 1.31 | 10.2 | 15.40 | 11.53 | 14.00 | 10.48 | 5230.77 | 3916.47 | 15.68 | 0 | 0.00 | 15 | 11.23 | 1 | 0.75 | 0 | 0.00 | 41 | 30.70 | 0 | 0.00 |
| 2 | 1 | Control | 4.85 | 257.32 | 3.17 | 1.54 | 1.32 | 10.2 | 4.68 | 3.05 | 3.90 | 2.54 | 1516.59 | 987.39 | 14.49 | 1 | 0.65 | 5 | 3.26 | 1 | 0.65 | 0 | 0.00 | 26 | 16.93 | 0 | 0.00 |
| 2 | 2 | Control | 3.79 | 238.22 | 3.13 | 1.19 | 1.30 | 10 | 4.76 | 4.01 | 4.40 | 3.70 | 1730.74 | 1457.32 | 15.34 | 6 | 5.05 | 2 | 1.68 | 0 | 0.00 | 1 | 0.84 | 8 | 6.74 | 0 | 0.00 |
| 2 | 3 | Control | 4.88 | 261.43 | 3.19 | 1.55 | 1.33 | 10.1 | 5.40 | 3.48 | 4.10 | 2.64 | 1542.70 | 993.22 | 14.63 | 0 | 0.00 | 5 | 3.22 | 0 | 0.00 | 0 | 0.00 | 14 | 9.01 | 0 | 0.00 |
| 2 | 4 | Control | 5.05 | 263.27 | 3.19 | 1.61 | 1.33 | 10 | 7.20 | 4.48 | 6.24 | 3.88 | 2216.87 | 1378.10 | 16.03 | 1 | 0.62 | 3 | 1.86 | 0 | 0.00 | 0 | 0.00 | 18 | 11.19 | 0 | 0.00 |
| 2 | 5 | Control | 4.31 | 251.65 | 3.26 | 1.41 | 1.36 | 9.8 | 5.45 | 3.88 | 4.50 | 3.20 | 1783.71 | 1269.52 | 14.30 | 2 | 1.42 | 5 | 3.56 | 0 | 0.00 | 0 | 0.00 | 11 | 7.83 | 0 | 0.00 |
| 2 | 6 | Control | 4.16 | 264.86 | 3.24 | 1.35 | 1.35 | 10.5 | 15.80 | 11.73 | 14.30 | 10.62 | 5677.00 | 4215.12 | 14.37 | 2 | 1.48 | 6 | 4.45 | 1 | 0.74 | 0 | 0.00 | 33 | 24.50 | 1 | 0.74 |
| 3 | 1 | Control | 4.08 | 277.29 | 3.54 | 1.44 | 1.47 | 11.3 | 18.38 | 12.74 | 15.30 | 10.60 | 5608.66 | 3887.20 | 15.76 | 13 | 9.01 | 14 | 9.70 | 48 | 33.27 | 22 | 15.25 | 28 | 19.41 | 8 | 5.54 |
| 3 | 2 | Control | 4.74 | 210.92 | 3.20 | 1.52 | 1.33 | 9.7 | 3.52 | 2.32 | 0.42 | 0.28 | 154.17 | 101.52 | 14.81 | 2 | 1.32 | 6 | 3.95 | 116 | 76.38 | 8 | 5.27 | 8 | 5.27 | 10 | 6.58 |
| 3 | 3 | Control | 4.56 | 236.94 | 3.35 | 1.53 | 1.40 | 9.3 | 2.52 | 1.65 | 1.16 | 0.76 | 442.28 | 289.27 | 14.64 | 3 | 1.96 | 10 | 6.54 | 8 | 5.23 | 0 | 0.00 | 30 | 19.62 | 1 | 0.65 |
| 3 | 4 | Control | 3.66 | 276.96 | 3.20 | 1.17 | 1.33 | 9.7 | 2.64 | 2.25 | 1.28 | 1.09 | 453.68 | 387.18 | 14.97 | 4 | 3.41 | 3 | 2.56 | 24 | 20.48 | 2 | 1.71 | 6 | 5.12 | 3 | 2.56 |
| 3 | 5 | Control | 4.25 | 247.00 | 2.85 | 1.21 | 1.19 | 9.6 | 18.90 | 15.59 | 16.80 | 13.86 | 5861.64 | 4835.81 | 16.13 | 3 | 2.47 | 12 | 9.90 | 8 | 6.60 | 17 | 14.02 | 23 | 18.97 | 6 | 4.95 |
| 3 | 6 | Control | 4.16 | 274.50 | 3.76 | 1.56 | 1.57 | 12.4 | 16.54 | 10.58 | 13.50 | 8.64 | 4300.71 | 2751.74 | 15.53 | 4 | 2.56 | 3 | 1.92 | 4 | 2.56 | 17 | 10.88 | 8 | 5.12 | 5 | 3.20 |
| 4 | 1 | Control | 4.01 | 263.03 | 3.35 | 1.34 | 1.40 | 9.8 | 14.92 | 11.10 | 6.50 | 4.84 | 2173.34 | 1616.83 | 15.28 | 16 | 11.90 | 250 | 185.98 | 48 | 35.71 | 0 | 0.00 | 112 | 83.32 | 22 | 16.37 |
| 4 | 2 | Control | 4.77 | 245.34 | 3.26 | 1.56 | 1.36 | 10 | 15.60 | 10.03 | 10.70 | 6.88 | 3548.54 | 2281.64 | 16.39 | 16 | 10.29 | 23 | 14.79 | 0 | 0.00 | 11 | 7.07 | 25 | 16.07 | 1 | 0.64 |
| 4 | 3 | Control | 4.54 | 245.61 | 3.20 | 1.45 | 1.33 | 10.3 | 14.00 | 9.63 | 8.50 | 5.85 | 2988.09 | 2055.67 | 16.05 | 19 | 13.07 | 40 | 27.52 | 3 | 2.06 | 15 | 10.32 | 19 | 13.07 | 0 | 0.00 |
| 4 | 4 | Control | 4.06 | 269.57 | 3.26 | 1.32 | 1.36 | 10.1 | 10.68 | 8.07 | 6.80 | 5.14 | 2415.61 | 1824.50 | 16.04 | 10 | 7.55 | 43 | 32.48 | 0 | 0.00 | 21 | 15.86 | 47 | 35.50 | 6 | 4.53 |
| 4 | 5 | Control | 4.76 | 264.85 | 3.28 | 1.56 | 1.37 | 9.8 | 29.00 | 18.60 | 24.00 | 15.40 | 7609.02 | 4881.05 | 15.64 | 2 | 1.28 | 96 | 61.58 | 0 | 0.00 | 12 | 7.70 | 30 | 19.24 | 2 | 1.28 |
| 4 | 6 | Control | 3.92 | 261.88 | 3.28 | 1.28 | 1.37 | 9.9 | 39.54 | 30.78 | 33.80 | 26.31 | 12315.95 | 9586.79 | 15.87 | 5 | 3.89 | 53 | 41.26 | 1 | 0.78 | 11 | 8.56 | 20 | 15.57 | 4 | 3.11 |
| 5 | 1 | Control | 4.41 | 261.44 | 3.70 | 1.63 | 1.54 | 10.6 | 2.00 | 1.22 | 0.12 | 0.07 | 37.55 | 22.98 | 16.69 | 17 | 10.40 | 13 | 7.96 | 1 | 0.61 | 27 | 16.53 | 5 | 3.06 | 21 | 12.85 |
| 5 | 2 | Control | 4.52 | 242.86 | 3.00 | 1.36 | 1.25 | 10.7 | 3.84 | 2.83 | 0.30 | 0.22 | 111.39 | 82.12 | 15.86 | 14 | 10.32 | 22 | 16.22 | 3 | 2.21 | 14 | 10.32 | 8 | 5.90 | 16 | 11.80 |
| 5 | 3 | Control | 3.99 | 278.39 | 3.32 | 1.32 | 1.38 | 10.9 | 1.74 | 1.32 | 0.52 | 0.39 | 218.46 | 165.34 | 14.11 | 13 | 9.84 | 6 | 4.54 | 7 | 5.30 | 0 | 0.00 | 7 | 5.30 | 1 | 0.76 |
| 5 | 4 | Control | 4.10 | 270.84 | 3.26 | 1.34 | 1.36 | 10.3 | 2.24 | 1.68 | 1.70 | 1.27 | 658.77 | 492.95 | 15.37 | 3 | 2.24 | 2 | 1.50 | 2 | 1.50 | 0 | 0.00 | 12 | 8.98 | 0 | 0.00 |
| 5 | 5 | Control | 4.36 | 274.50 | 3.41 | 1.49 | 1.42 | 10.5 | 0.84 | 0.56 | 0.10 | 0.07 | 42.80 | 28.78 | 15.24 | 3 | 2.02 | 6 | 4.03 | 7 | 4.71 | 3 | 2.02 | 11 | 7.40 | 2 | 1.34 |
| 5 | 6 | Control | 4.60 | 266.93 | 3.30 | 1.52 | 1.37 | 10.4 | 1.08 | 0.71 | 0.14 | 0.09 | 59.04 | 38.93 | 14.05 | 3 | 1.98 | 7 | 4.62 | 5 | 3.30 | 7 | 4.62 | 14 | 9.23 | 6 | 3.96 |
| 1 | 1 | Treatment | 4.02 | 263.79 | 3.20 | 1.29 | 1.33 | 9.7 | 14.10 | 10.94 | 13.40 | 10.39 | 5118.00 | 3969.87 | 15.57 | 0 | 0.00 | 4 | 3.10 | 0 | 0.00 | 0 | 0.00 | 4 | 3.10 | 0 | 0.00 |
| 1 | 2 | Treatment | 4.46 | 265.76 | 3.20 | 1.43 | 1.33 | 10.2 | 12.36 | 8.66 | 11.50 | 8.05 | 4776.28 | 3344.62 | 15.12 | 1 | 0.70 | 7 | 4.90 | 0 | 0.00 | 1 | 0.70 | 8 | 5.60 | 0 | 0.00 |
| 2 | 1 | Treatment | 4.19 | 255.31 | 3.17 | 1.33 | 1.32 | 10.2 | 5.00 | 3.77 | 4.30 | 3.24 | 1687.34 | 1271.61 | 15.33 | 4 | 3.01 | 15 | 11.30 | 1 | 0.75 | 0 | 0.00 | 22 | 16.58 | 1 | 0.75 |
| 2 | 2 | Treatment | 4.89 | 251.44 | 3.13 | 1.53 | 1.30 | 10 | 5.82 | 3.80 | 4.88 | 3.19 | 1941.07 | 1268.25 | 15.31 | 6 | 3.92 | 4 | 2.61 | 0 | 0.00 | 0 | 0.00 | 19 | 12.41 | 1 | 0.65 |
| 2 | 3 | Treatment | 3.84 | 268.39 | 3.19 | 1.22 | 1.33 | 10.1 | 5.20 | 4.25 | 4.40 | 3.60 | 1735.69 | 1418.97 | 15.38 | 0 | 0.00 | 6 | 4.91 | 0 | 0.00 | 2 | 1.64 | 13 | 10.63 | 0 | 0.00 |
| 2 | 4 | Treatment | 3.97 | 262.72 | 3.19 | 1.26 | 1.33 | 10 | 5.60 | 4.43 | 4.60 | 3.64 | 1783.30 | 1411.34 | 15.48 | 0 | 0.00 | 3 | 2.37 | 0 | 0.00 | 0 | 0.00 | 9 | 7.12 | 0 | 0.00 |
| 2 | 5 | Treatment | 4.59 | 262.23 | 3.26 | 1.50 | 1.36 | 9.8 | 6.70 | 4.48 | 5.30 | 3.54 | 2315.29 | 1547.37 | 14.79 | 3 | 2.00 | 6 | 4.01 | 0 | 0.00 | 0 | 0.00 | 39 | 26.06 | 0 | 0.00 |
| 2 | 6 | Treatment | 4.45 | 255.67 | 3.24 | 1.44 | 1.35 | 10.5 | 15.18 | 10.52 | 13.80 | 9.56 | 5770.92 | 3999.21 | 15.04 | 4 | 2.77 | 3 | 2.08 | 0 | 0.00 | 0 | 0.00 | 9 | 6.24 | 0 | 0.00 |
| 3 | 1 | Treatment | 4.07 | 258.27 | 3.54 | 1.44 | 1.47 | 11.3 | 17.92 | 12.46 | 16.10 | 11.19 | 6358.13 | 4419.94 | 15.43 | 7 | 4.87 | 11 | 7.65 | 20 | 13.90 | 11 | 7.65 | 12 | 8.34 | 3 | 2.09 |
| 3 | 2 | Treatment | 3.97 | 256.50 | 3.20 | 1.27 | 1.33 | 9.7 | 1.76 | 1.39 | 0.30 | 0.24 | 108.08 | 85.08 | 16.01 | 1 | 0.79 | 20 | 15.74 | 1 | 0.79 | 16 | 12.59 | 5 | 3.94 | 3 | 2.36 |
| 3 | 3 | Treatment | 4.20 | 263.32 | 3.35 | 1.41 | 1.40 | 9.3 | 2.28 | 1.62 | 1.24 | 0.88 | 485.13 | 344.58 | 15.40 | 1 | 0.71 | 9 | 6.39 | 9 | 6.39 | 0 | 0.00 | 25 | 17.76 | 0 | 0.00 |
| 3 | 4 | Treatment | 4.17 | 267.50 | 3.20 | 1.34 | 1.33 | 9.7 | 2.22 | 1.66 | 1.34 | 1.00 | 490.64 | 367.37 | 15.78 | 2 | 1.50 | 4 | 3.00 | 11 | 8.24 | 5 | 3.74 | 7 | 5.24 | 3 | 2.25 |
| 3 | 5 | Treatment | 3.36 | 263.77 | 2.85 | 0.96 | 1.19 | 9.6 | 12.14 | 12.67 | 10.50 | 10.96 | 3415.21 | 3563.83 | 16.41 | 5 | 5.22 | 9 | 9.39 | 7 | 7.30 | 8 | 8.35 | 9 | 9.39 | 3 | 3.13 |
| 3 | 6 | Treatment | 4.34 | 257.53 | 3.76 | 1.63 | 1.57 | 12.4 | 18.30 | 11.22 | 14.90 | 9.13 | 5313.60 | 3256.58 | 16.02 | 3 | 1.84 | 7 | 4.29 | 5 | 3.06 | 14 | 8.58 | 19 | 11.64 | 5 | 3.06 |
| 4 | 1 | Treatment | 4.40 | 230.97 | 3.35 | 1.47 | 1.40 | 9.8 | 12.20 | 8.27 | 6.40 | 4.34 | 2376.61 | 1611.34 | 15.76 | 16 | 10.85 | 64 | 43.39 | 4 | 2.71 | 52 | 35.26 | 72 | 48.82 | 20 | 13.56 |
| 4 | 2 | Treatment | 3.71 | 278.39 | 3.26 | 1.21 | 1.36 | 10 | 17.58 | 14.55 | 7.60 | 6.29 | 2565.13 | 2123.56 | 16.27 | 17 | 14.07 | 40 | 33.11 | 0 | 0.00 | 19 | 15.73 | 7 | 5.79 | 4 | 3.31 |
| 4 | 3 | Treatment | 3.66 | 262.81 | 3.20 | 1.17 | 1.33 | 10.3 | 14.22 | 12.13 | 8.60 | 7.34 | 3207.72 | 2737.03 | 15.77 | 24 | 20.48 | 54 | 46.08 | 1 | 0.85 | 22 | 18.77 | 0 | 0.00 | 2 | 1.71 |
| 4 | 4 | Treatment | 4.79 | 240.91 | 3.26 | 1.56 | 1.36 | 10.1 | 7.80 | 5.00 | 5.00 | 3.21 | 1777.43 | 1139.61 | 15.97 | 13 | 8.34 | 21 | 13.46 | 0 | 0.00 | 13 | 8.34 | 10 | 6.41 | 3 | 1.92 |
| 4 | 5 | Treatment | 3.58 | 257.45 | 3.28 | 1.17 | 1.37 | 9.8 | 31.04 | 26.45 | 28.00 | 23.86 | 10460.73 | 8913.83 | 15.74 | 8 | 6.82 | 57 | 48.57 | 0 | 0.00 | 33 | 28.12 | 26 | 22.16 | 1 | 0.85 |
| 4 | 6 | Treatment | 4.39 | 264.29 | 3.28 | 1.44 | 1.37 | 9.9 | 29.50 | 20.50 | 27.00 | 18.77 | 9077.44 | 6309.50 | 16.32 | 3 | 2.09 | 18 | 12.51 | 0 | 0.00 | 9 | 6.26 | 13 | 9.04 | 4 | 2.78 |
| 5 | 1 | Treatment | 3.61 | 273.24 | 3.70 | 1.34 | 1.54 | 10.6 | 2.62 | 1.96 | 0.12 | 0.09 | 37.71 | 28.20 | 16.67 | 19 | 14.21 | 16 | 11.97 | 6 | 4.49 | 18 | 13.46 | 7 | 5.24 | 19 | 14.21 |
| 5 | 2 | Treatment | 3.55 | 255.77 | 3.00 | 1.06 | 1.25 | 10.7 | 1.88 | 1.77 | 0.18 | 0.17 | 68.17 | 64.09 | 15.74 | 6 | 5.64 | 1 | 0.94 | 5 | 4.70 | 30 | 28.21 | 0 | 0.00 | 4 | 3.76 |
| 5 | 3 | Treatment | 4.48 | 259.32 | 3.32 | 1.48 | 1.38 | 10.9 | 1.66 | 1.12 | 0.50 | 0.34 | 201.07 | 135.54 | 15.31 | 6 | 4.04 | 10 | 6.74 | 0 | 0.00 | 0 | 0.00 | 8 | 5.39 | 1 | 0.67 |
| 5 | 4 | Treatment | 4.70 | 238.64 | 3.26 | 1.53 | 1.36 | 10.3 | 2.34 | 1.53 | 1.60 | 1.05 | 609.03 | 397.97 | 15.47 | 9 | 5.88 | 7 | 4.57 | 0 | 0.00 | 0 | 0.00 | 4 | 2.61 | 1 | 0.65 |
| 5 | 5 | Treatment | 4.40 | 258.01 | 3.41 | 1.50 | 1.42 | 10.5 | 0.82 | 0.55 | 0.10 | 0.07 | 41.47 | 27.66 | 15.34 | 3 | 2.00 | 5 | 3.33 | 7 | 4.67 | 4 | 2.67 | 3 | 2.00 | 3 | 2.00 |
| 5 | 6 | Treatment | 3.38 | 278.30 | 3.30 | 1.11 | 1.37 | 10.4 | 0.82 | 0.74 | 0.16 | 0.14 | 54.24 | 48.66 | 16.35 | 5 | 4.49 | 3 | 2.69 | 3 | 2.69 | 2 | 1.79 | 8 | 7.18 | 6 | 5.38 |
